# Perception of Upright: Multisensory Convergence and the Role of Temporo-Parietal Cortex

**DOI:** 10.3389/fneur.2017.00552

**Published:** 2017-10-25

**Authors:** Amir Kheradmand, Ariel Winnick

**Affiliations:** ^1^Department of Neurology, The Johns Hopkins University School of Medicine, Baltimore, MD, United States; ^2^Department of Otolaryngology – Head and Neck Surgery, The Johns Hopkins University School of Medicine, Baltimore, MD, United States

**Keywords:** subjective visual vertical, cerebral cortex, upright perception, Bayesian, temporo-parietal cortex, spatial orientation, orientation constancy, ocular torsion

## Abstract

We inherently maintain a stable perception of the world despite frequent changes in the head, eye, and body positions. Such “orientation constancy” is a prerequisite for coherent spatial perception and sensorimotor planning. As a multimodal sensory reference, perception of upright represents neural processes that subserve orientation constancy through integration of sensory information encoding the eye, head, and body positions. Although perception of upright is distinct from perception of body orientation, they share similar neural substrates within the cerebral cortical networks involved in perception of spatial orientation. These cortical networks, mainly within the temporo-parietal junction, are crucial for multisensory processing and integration that generate sensory reference frames for coherent perception of self-position and extrapersonal space transformations. In this review, we focus on these neural mechanisms and discuss (i) neurobehavioral aspects of orientation constancy, (ii) sensory models that address the neurophysiology underlying perception of upright, and (iii) the current evidence for the role of cerebral cortex in perception of upright and orientation constancy, including findings from the neurological disorders that affect cortical function.

## Introduction

Spatial orientation refers to the perceptual awareness of the body position relative to the environment. While oriented to the surroundings, we maintain a stable perception of the world in upright orientation despite frequent changes in the eye, head, and body positions. Such “orientation constancy” is a key functional aspect of our spatial perception, and if disrupted the consequences can be quite debilitating due to ensuing dizziness, disorientation, and loss of balance. These symptoms are often triggered by motion or changes in the head or body positions, e.g., as in patients with vestibular dysfunction. Our perception of spatial orientation is possible because the position of the body is linked to the external environment through processing and integration of visual, vestibular, and proprioceptive information. In this process, the compensatory movement of the eyes through the vestibulo-ocular reflex is vital to maintain visual stability with changes in the head position. In frontal-eyed animals, in addition to the horizontal and vertical eye movements, lateral head tilts (i.e., with respect to gravity) lead to changes in the torsional eye position in the opposite direction of the head tilt. In humans, this ocular counter-roll (OCR) is a constrained, phylogenetically old vestibular reflex and does not match the magnitude of the head tilt (1). Such “visual-vestibular” mismatch, although sounds counter-productive, may actually represent an evolutionary advantage, as it can provide the brain with pertinent cues to quickly deconstruct perceived tilts into changes in the body position and the visual world, thus facilitating interactions with the surrounding environment. In this scheme, however, to achieve orientation constancy, the brain must be able to generate a common reference frame based on the sensory inputs that are inevitably encoded in different reference frames.

**Figure 1:**
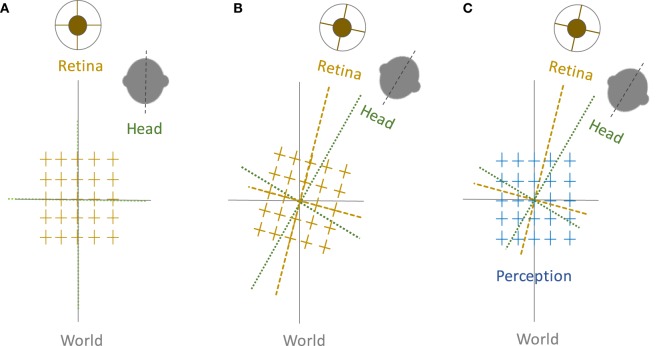
Perception of upright and sensory reference frames: The head, eye, and the world reference frames are all aligned in upright position along the gravitational vertical **(A)**, but when the head is tilted, the ocular counter-roll only partially compensates for the amount of head tilt (gain about 10–25%), which results in a separation of the sensory reference frames that encode head-in-space and eye/retina-in-head orientations **(B)**. Despite these differences, visual perception remains in upright orientation **(C)**. Therefore, the brain—like any other sensorimotor system—must be able to integrate sensory inputs into a common reference frame to maintain a coherent perception of upright.

Let us examine a simple lateral head tilt more closely. In the upright position—where the vertical meridians of the eyes, head, body, and the visual world are all aligned with the gravitational vertical—maintaining upright perception is not challenging for the brain. However, as mentioned earlier, when the head is tilted and as the brain senses changes in the head position relative to gravity, OCR will only partially compensate for the amount of head tilt, typically with a low gain of about 10–25% in humans. Therefore, as a result of head tilt, the reference frames for the head, eye, and the visual world are no longer aligned along the gravitational vertical, and images become tilted on the retina (Figure 1). Despite separation of these individual sensory reference frames, our visual perception remains in upright orientation within a common reference frame. This perceptual constancy in upright orientation can be effectively studied by removing orienting visual cues, in which case the brain has to rely on information about the head and body positions in space and the eye position in the orbit to determine the orientation of external stimuli. A similar approach has been the basis of psychophysical experiments dating back to 1860. Around that time, Hermann Aubert, an expert in optics, used afterimages to investigate perception of vertical and horizontal line orientations in light and darkness. Using afterimages of a bright line, Aubert tilted his head with eyes closed until the afterimage was earth-horizontal. Upon opening the eyes, he found that the afterimage would deviate toward the side of the head tilt (2). George Elias Müller then investigated a range of smaller head tilts and found that the line would deviate away from the side of the head tilt (3). Müller also put forth theories to describe these perceptual errors, considering sensory contributions from the otoliths, semicircular canals, and proprioception (3). Later on, mathematical models were used to account for these findings. One of the initial quantitative models was put forth by Mittelstaedt in 1983, in which he proposed that the brain must generate an internal common reference to “*stabilize man’s confidence in the stability of his world*” (4). From this perspective, he eloquently posited about discrepancies between the elements of our perception and the real world:

… in this facet of his subjectivity, man appears as a creature, whose mind underrates the humble services of his bodily feelings while naively taking at face value what [he] believes to see, unaware of being deceived, as it were, by the workings of a machinery which toils in the interest of survival but not in the service of truth… (4).

In recent decades, contributions of various sensory modalities to perception of upright have been studied extensively. However, currently, less is known about the neural structures and functions involved in orientation constancy. In this review, we first focus on neurobehavioral aspects of orientation constancy and describe sensory models that address the neurophysiology underlying upright perception. We then review the current evidence for the role of cerebral cortex in perception of upright and orientation constancy. Finally, we outline findings from neurological disorders that impact cortical mechanisms underlying perception of upright.

## Neurobehavioral Aspects of Upright Perception

### Measurement Paradigms

**Figure 2:**
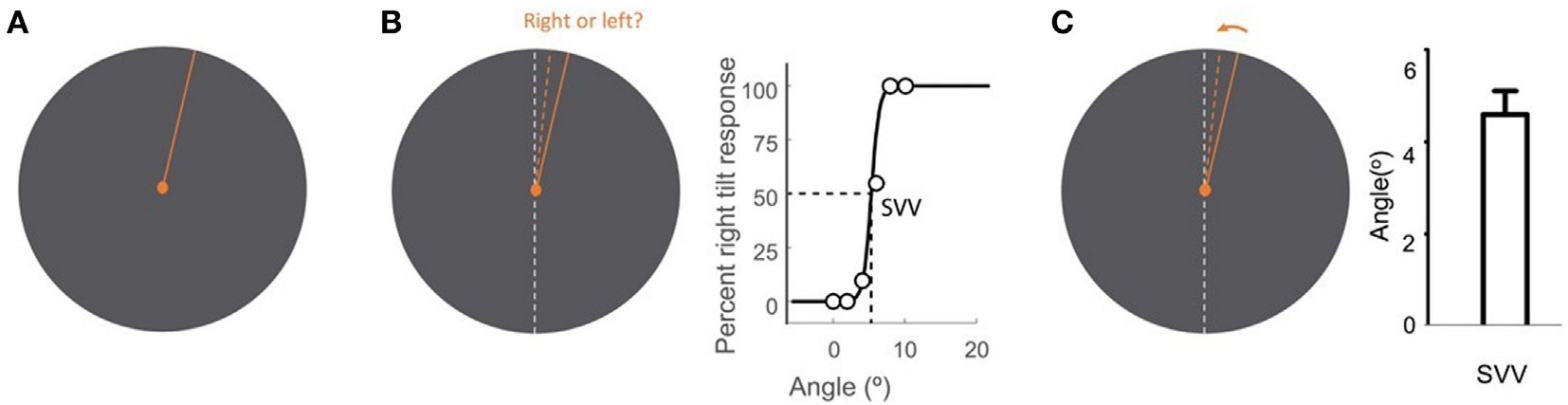
Subjective visual vertical (SVV) measurement with the line stimulus (solid orange) presented at a random orientation in each trial **(A)**. In the forced-choice paradigm, the task is to report whether the line is tilted to the right or to the left of the perceived upright orientation (dashed orange) **(B)**. SVV is then determined by fitting a psychometric curve to the responses from all trials and is calculated as the value on the curve at which the probability of left or right responses is 50% (point of subjective equality). In the active-adjustment paradigm, the line stimulus (solid orange) is adjusted (direction shown by arrow) to the perceived upright orientation (dashed orange) **(C)**. In this paradigm, SVV is calculated as the average value from all trials. The true vertical is shown by the dashed white line **(B,C)**.

Upright perception is typically studied by means of a psychophysical task known as the subjective visual vertical (SVV). In this task, a visual line is used to report perceived earth-vertical orientation in the absence of visual cues. Various methods have been described for SVV measurement. Some paradigms use active adjustment of the visual line stimulus, and others are based on a forced-choice task, where in each trial a visual line orientation is reported with respect to the perceived upright orientation (Figure 2).

Although the visual exposure in SVV paradigms is limited to a line stimulus without any other orienting cues, the line itself may affect SVV responses, especially during active adjustments (5–7). For example, the initial orientation of the line stimulus can bias upright perception in the direction of the starting line orientation, and in the opposite direction of the line movement (7–12). This bias, however, may reverse and occur as a “hysteresis” effect in the direction of the line movement when the line is presented in sequential angles in a forced-choice paradigm (6). Also, with active adjustment of the line, the upright estimate may gradually drift as a result of trial-to-trial dependency of upright adjustments and inter-correlation among consecutive SVV responses (13). In addition, the torsional position of the eyes can change in the direction of the visual line rotation, and such “torsional entrainment” may introduce biases when SVV is measured using the line rotation (14). Considering all these sources of error, a forced-choice task with a random line orientation in each trial would be the least biased method for SVV measurement, as it would remove the effects of line movement on SVV responses (15).

The length of the line stimulus can also influence SVV responses, resulting in biases in the direction of the body tilt with longer lines and in the opposite direction of the body tilt with shorter lines (16, 17). Another factor that can affect magnitude of SVV errors is the viewing distance from the visual line stimulus (18). This effect has been attributed to ocular torsion induced by changes in the vergence angle of the eyes (i.e., cycloversion). The viewing eye (i.e., monocular or binocular viewing), on the other hand, does not significantly affect SVV errors, neither in upright position nor during head tilt (6, 18).

### Systematic Errors

Subjective visual vertical errors reflect challenges for the brain in maintaining a common reference frame based on sensory information encoding eye, head, and body positions. In upright position, SVV errors typically remain within 2° of earth vertical (4, 19–21). However, with lateral head or whole body tilts, there are systematic errors in the perceived upright orientation which do not correspond with the errors in perception of body tilt (4, 19, 22–25). Such inherent dissociation between the perceptions of body tilt and upright orientation is also seen with active body tilts (as opposed to passive tilts), even when the brain has access to additional proprioceptive cues or efference copy signals to encode the veridical position of the body (23).

**Figure 3:**
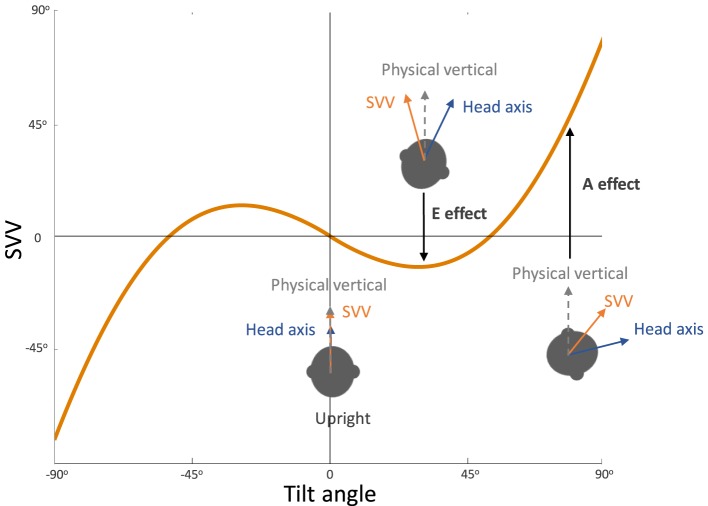
Systematic errors of subjective visual vertical (SVV): healthy individuals typically have SVV errors within 2° of earth vertical in upright position. At large tilt angles (usually greater than 60°), SVV errors are deviated toward the tilt direction, which reflect “underestimation” of upright orientation (known as the Aubert or A-effect). At smaller tilt angles (usually less than 60°), however, SVV errors are often opposite to the tilt direction, which reflect “overestimation” of upright orientation (known as the Entgegengesetzt or E-effect).

In general, SVV errors are biased toward the direction of the body position at tilt angles greater than 60°. This finding, which reflects underestimation of upright orientation, is known as the Aubert or A-effect (Figure 3) (2, 4, 19, 20). At smaller tilt angles (e.g., less than 60°), however, SVV errors are often biased in the opposite direction of the body position. This finding, which reflects overestimation of upright orientation, is known as the Müller or E-effect (E for “Entgegengesetzt,” German for opposite) (3, 19–21). The peak underestimation error of the A-effect is usually around 130°, and beyond this tilt angle the E-effect usually occurs again which is attributed to switching of the internal upright reference frame from the head to the feet (19, 21, 24, 26–28). Overall, the E-effect presents less consistently and less often compared with the A-effect (21, 24, 29). The variability of SVV responses also increases with the body tilts up to 120–150°, and then decreases again with the tilt angles approaching 180° (21, 26, 29–34). This pattern of SVV variability has been attributed to a tilt-dependent noise in the otolith and proprioceptive inputs (4, 21).

### Other Measurement Methods

Some studies have used subjective visual horizontal (SVH) instead of visual vertical measurements. The results, however, show that SVV and SVH are not invariably orthogonal to one another, especially at larger tilt angles (13, 18, 19, 35–37). In other words, errors of vertical and horizontal perception may not match at the same body tilt position and while SVV errors tend be larger in the direction of the tilt (i.e., SVV errors show larger A-effects), SVH errors tend to be larger in the opposite direction of the tilt (i.e., SVH errors show larger E-effects) (18). SVV and SVH errors have also been studied in the pitch plane, and—similar to the systematic errors in the roll plane—they reflect overestimations in the opposite direction of small pitch angles, and underestimations in the direction of large pitch angles (4, 38–40).

Another common method for measuring upright perception is with a haptic stimulus. Similar to SVV, haptic upright responses become less precise at large body tilt angles, but in some individuals they can be more accurate compared with the visual vertical responses (41–44). Also, haptic measurements tend to produce larger E-effects at smaller tilt angles (i.e., less than 60°) and may become more accurate in the supine position compared with the upright position (45–47). More importantly, the perceived haptic or postural upright can be dissociated from the visual perception of upright (48, 49). For example, while patients with unilateral vestibular loss showed significant SVV errors, their postural vertical adjustments were not different from the healthy controls (48). The disparity in the SVV and postural vertical responses in this study suggests different weights of sensory contributions to perception of upright depending on the method of measurement (e.g., haptic versus visual tasks) (49, 50). However, only few patients were included here, and the postural vertical was measured while sitting in a motor-driven chair and adjusting its orientation to the perceived upright position. In keeping with such distinct sensory contributions, haptic upright responses, in contrast to SVV, were more biased by the whole body tilt than just the head-on-body tilt in a group of healthy individuals (49).

### Spatial Perception Models

**Figure 4:**
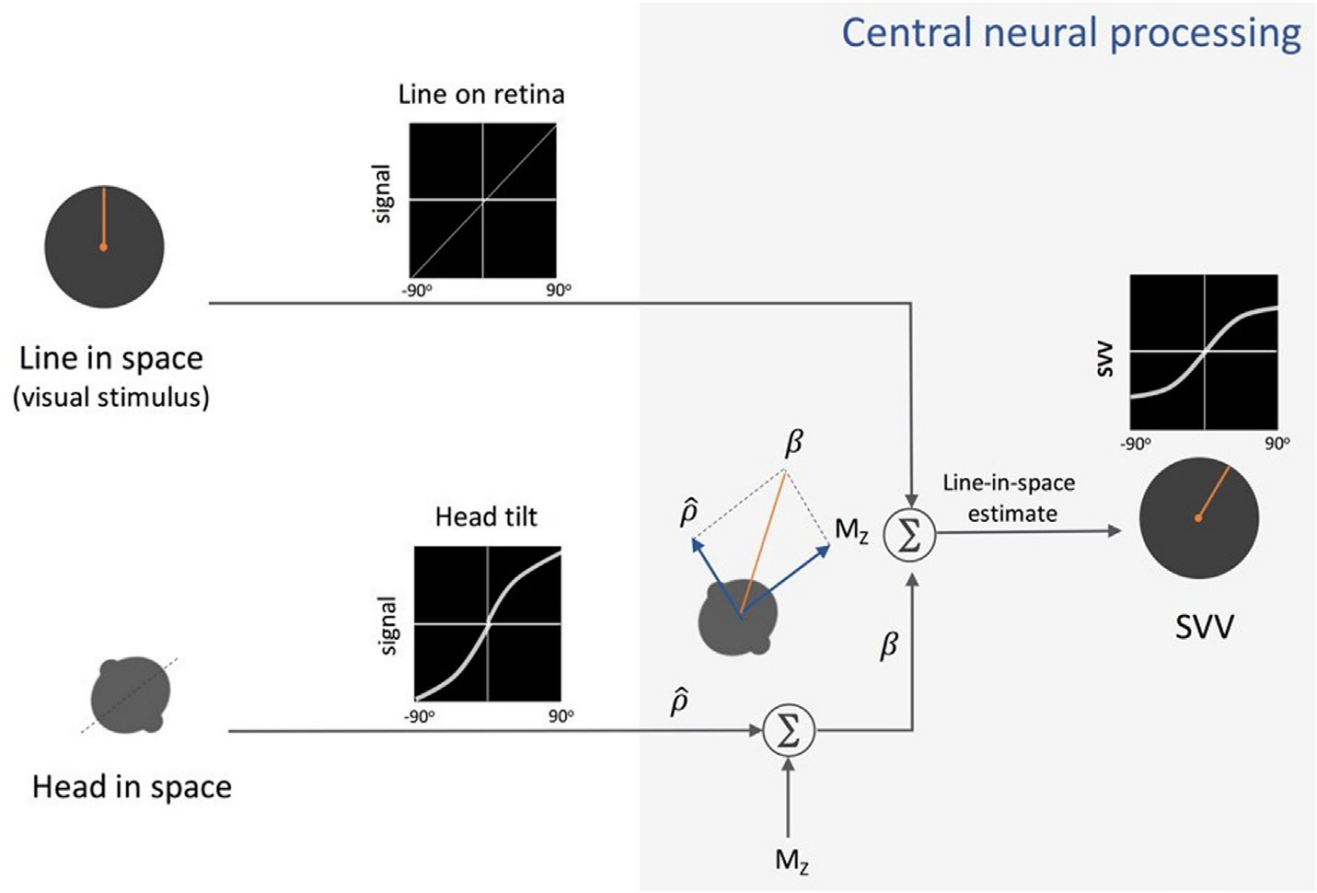
Schematic presentation of Mittelstaedt’s idiotropic vector model: visual line orientation on the retina and head tilt are the two sensory inputs in this model. The visual signal is accurate, but the head tilt signal $\left(\hat{\rho }\right)$ shows increasing errors with head tilt (a range of ±90° tilt is shown in black graphs). As part of the central neural processing, vectorial summation of the head tilt signal $\left(\hat{\rho }\right)$ and the head-fixed idiotropic vector (M_Z_) yields the compensatory tilt signal (β). The compensatory tilt signal and the visual signal are then added to obtain an internal estimate of the upright orientation [i.e., subjective visual vertical (SVV)].

In recent years, several studies have addressed neural mechanisms underlying perception of upright and the systematic errors with changes in body tilt orientation. Mittelstaedt first put forward a model in 1983 that could account for the A-effect (4). He proposed that the brain implements a computational strategy based on an internal bias signal to correct for the noisy inputs from the otolith organs (Figure 4). This internal signal, referred to as “the idiotropic vector,” is a constant, body-fixed vector that is added to the estimated direction of gravity from the otolith inputs to determine upright orientation. At large body tilts, the effect of idiotropic vector results in a bias in upright estimates toward the body axis and thus the A-effect. According to this model, the computation of upright orientation does not influence the estimate of body tilt. Therefore, the idiotropic vector could be viewed as a computational strategy to reduce distortions in upright perception for commonly encountered small body tilts, at the expense of large A-effects for rarely encountered large body tilts.

**Figure 5:**
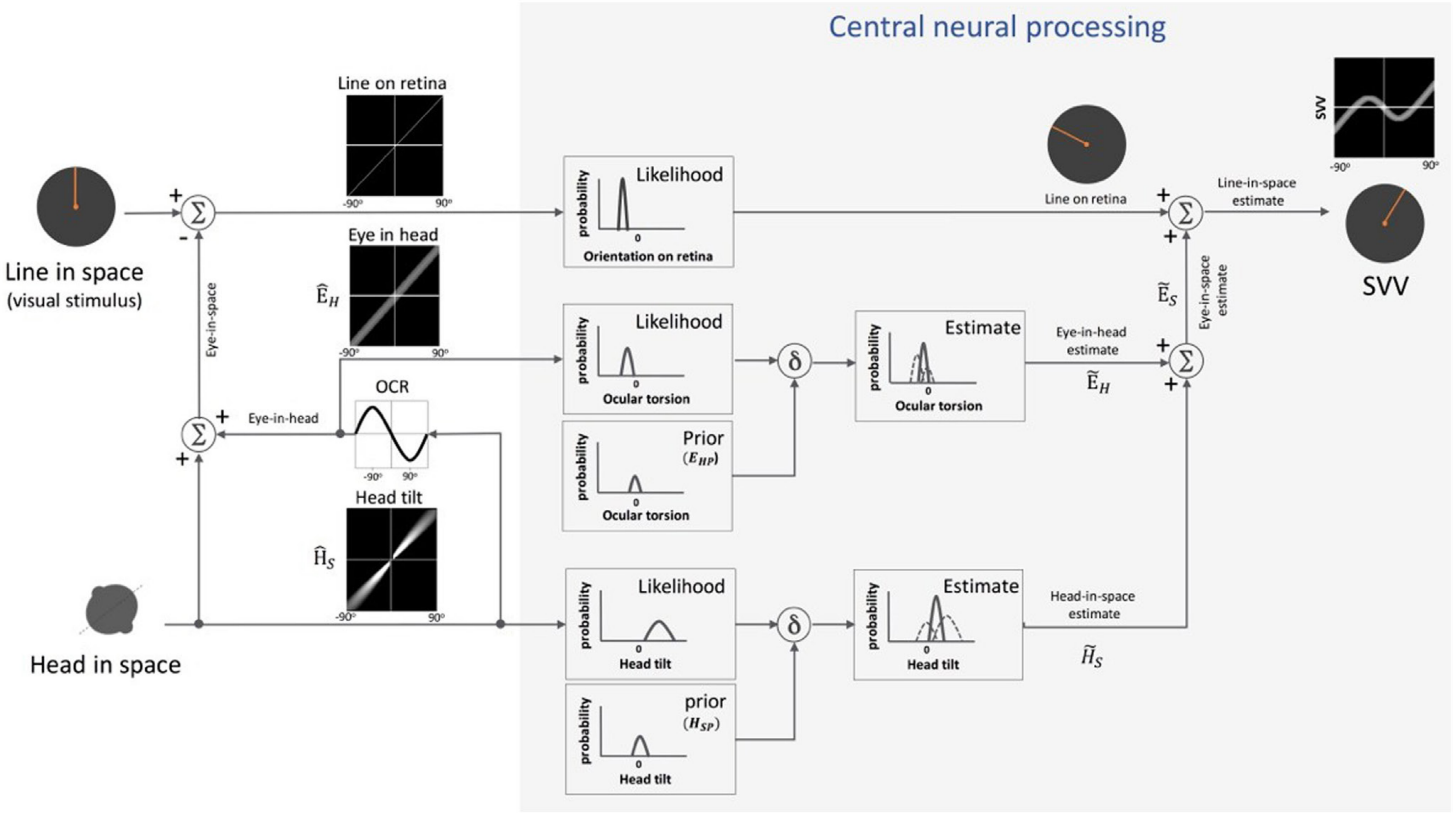
Schematic presentation of Bayesian spatial perception model: various sensory modalities are integrated into a common spatial reference frame to determine upright orientation. A vertical line (line in space) is presented in front of a tilted observer (head in space) (a range of ±90° tilt is shown in black graphs). Signal ${\hat{H}}_{S}$, encoding head orientation in space, is accurate but contaminated by Gaussian noise. Head tilt results in ocular counter-roll (OCR) and signal ${\hat{E}}_{H}$, encoding eye-in-head orientation, is also contaminated by independent noise. As part of central neural processing, the estimates of head-in-space $\left({\tilde{H}}_{S}\right)$ and eye-in-head $\left({\tilde{E}}_{H}\right)$ are generated separately from the likelihoods and priors of head tilt and torsional eye position (i.e., ocular torsion). These estimates are integrated to generate eye-in-space estimate $\left({\tilde{E}}_{S}\right)$, which is then integrated with retinal signal (line on retina) to obtain an internal estimate of the upright orientation [i.e., subjective visual vertical (SVV)].

The effect of the idiotropic vector was later described within a Bayesian framework and was equated to the role of the Bayesian “prior” for processing noisy sensory signals (21, 29, 51–54). In this Bayesian spatial perception model, the upright estimate is determined by a weighted average of the existing knowledge of tilt position (i.e., the prior) and the likelihood of change in tilt position based on noisy sensory information (Figure 5). Since we spend most of our time in upright position, the prior for tilt position is a Gaussian distribution centered at 0° (i.e., upright position). Thus, the effect of prior could bias upright estimates and result in underestimation of true vertical at large tilt angles (i.e., the A-effect). According to the Bayesian model, the head estimate can be determined in the following relation (53):

(1) $${\tilde{H}}_{S}=\frac{\sigma_{H_{\mathrm{Sp}}}^{2}}{\sigma_{H_{\mathrm{Sp}}}^{2}+\sigma_{\hat{H_{S}}}^{2}}\cdot H_{S}.$$

In Eq. 1, ${\tilde{H}}_{S}$ represents the final head-in-space estimate by the brain (i.e., “the posterior” in Bayesian terms), ${\hat{H}}_{S}$ the head orientation in space as measured by the head-in-space sensors, and *H_S_* the actual head-in-space position (i.e., measured head position with respect to the direction of gravity). Among the sensory signals in the model, the head-in-space input $\text{(}{\hat{H}}_{S}\text{)}$ is noisy (with a variance of $\sigma_{\hat{H_{S}}}^{2}$), and thus the prior (with a small variance of $\sigma_{H_{\mathrm{Sp}}}^{2}$) is taken into account to estimate the final head position $\left({\tilde{H}}_{S}\right)$. Based on Eq. 1, the error in head estimate $\left(\mu_{\tilde{H_{S}}}\right)$ is given by:

(2) $$\mu_{{\tilde{H}}_{S}}=H_{S}-{\tilde{H}}_{S}=H_{S}-\frac{\sigma_{H_{\mathrm{Sp}}}^{2}}{\sigma_{H_{\mathrm{Sp}}}^{2}+\sigma_{\hat{H_{S}}}^{2}}\cdot H_{S}=\frac{\sigma_{\hat{H_{\text{S}}}}^{2}}{\sigma_{H_{\mathrm{Sp}}}^{2}+\sigma_{\hat{H_{S}}}^{2}}\cdot H_{S}.$$

De Vrijer et al. added another parameter to the Bayesian model to account for the error in estimating ocular torsion position by the brain $\left(\mu_{\tilde{E_{H}}}\right)$ (Figure 5) (53). This “uncompensated” ocular torsion can explain the SVV error in the opposite direction of the head tilt at smaller tilt angles (i.e., the E-effect). The error in estimating ocular torsion $\left(\mu_{\tilde{E_{H}}}\right)$ is determined in the following relation:

(3) $$\mu_{\tilde{E_{H}}}=\frac{\sigma_{\hat{E_{H}}}^{2}}{\sigma_{E_{Hp}}^{2}+\sigma_{\hat{E_{H}}}^{2}}\cdot \text{A sin}(H_{S}).$$

In this Eq. 3, *ÊH* is the eye-in-head position based on sensory inputs encoding ocular torsion and *E_HP_* is the prior for the eye-in-head position (with a variance $\sigma_{E_{Hp}}^{2}$), which is taken into account by the brain to estimate torsional eye position $\left({\tilde{E}}_{H}\right)$. The maximum torsion amplitude is denoted by A. Since the eyes always roll in the opposite direction of the head tilt, the final error in upright perception (μ_SVV_) can be given by subtracting Eqs 2 and 3 as below:

(4) $$\mu_{\text{SVV}}=\mu_{\tilde{H_{S}}}-\mu_{\tilde{E_{H}}}=\frac{\sigma_{\hat{H_{S}}}^{2}}{\sigma_{H_{\mathrm{Sp}}}^{2}+\sigma_{\hat{H_{S}}}^{2}}\cdot H_{S}-\frac{\sigma_{\hat{E_{H}}}^{2}}{\sigma_{E_{\mathrm{Hp}}}^{2}+\sigma_{\hat{E_{H}}}^{2}}\cdot \text{A sin}(H_{S}).$$

**Figure 6:**
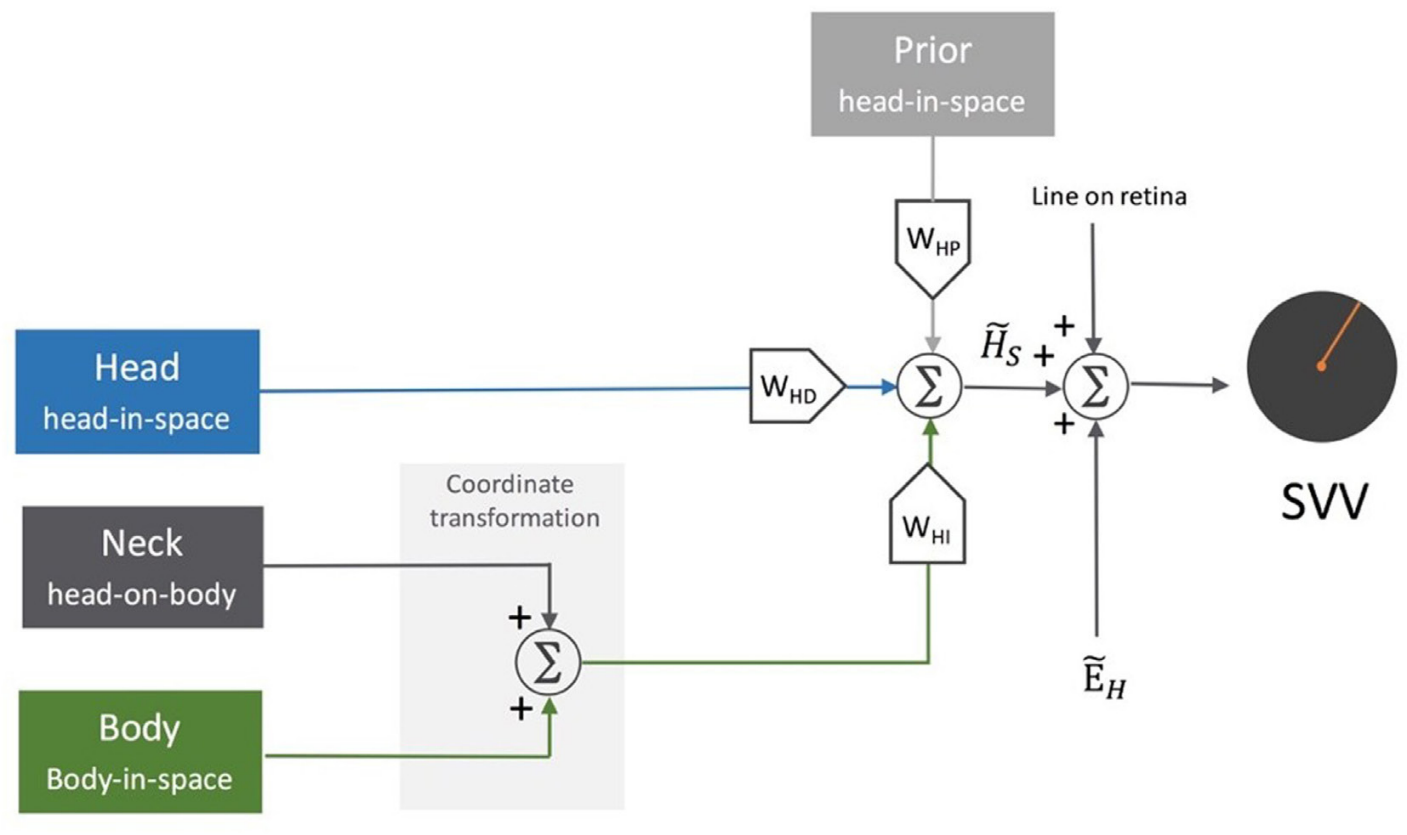
Schematic representation of the sensory integration model: body sensors, neck sensors, and otoliths provide information about the body in-space, head-on-body, and head-in-space positions, respectively. As part of the central neural processing, the neck and body signals undergo coordinate transformation to indirectly encode head-in-space orientation. Overall, the optimal head-in-space estimate $\left({\tilde{H}}_{S}\right)$ is obtained by the relative weights of the otolith information (*W_HD_*, blue pathway), coordinate-transformed information from the body and neck sensors (*W_HI_*, green pathway), and the head prior information (*W_HP_*, gray pathway). The head-in-space estimate $\left({\tilde{H}}_{S}\right)$ is then integrated with eye-in-head estimate $\left({\tilde{E}}_{H}\right)$ and line orientation on the retina to obtain an internal estimate of the upright orientation [i.e., subjective visual vertical (SVV)].

Since this model assumes a vertical orientation of the trunk, the estimate of head-in-space $\left({\hat{H}}_{S}\right)$ represents a combination of the otolith and proprioceptive inputs (53). Clemens et al. later proposed an update to separately account for the head and body positions using the following signals: the head orientation with respect to gravity (otoliths), body orientation in space (body proprioceptors), and the relative position of the head and body (neck proprioceptors) (54) (Figure 6). In this model, based on the optimal observer theory, the body orientation in space can be determined either “directly” using proprioceptive information from the trunk graviceptors (55–57) or “indirectly” from subtracting the signals encoding head and neck positions. Likewise, the estimate of head-in-space orientation can be obtained directly from the head position or indirectly from the body and neck proprioceptive signals. Accordingly, the optimal estimate of upright orientation is determined by integrating (1) direct information from the head position sensors (i.e., otoliths), (2) indirect information from the body and neck proprioceptors, and (3) prior information about the head and body orientations in space. The indirect sensory signals require reference frame transformation before integration with other sensory information. Thus, altogether, the final error in upright perception is calculated based on the weights of the direct and indirect information and is given by the following relation:

(5) $$\mu_{\text{SVV}}=\left(1-W_{\mathrm{HD}}-W_{\mathrm{HI}}\right)\cdot H_{S}-\frac{\sigma_{\hat{E_{H}}}^{2}}{\sigma_{E_{\mathrm{Hp}}}^{2}+\sigma_{\hat{E_{H}}}^{2}}\cdot \text{A sin}(H_{S}).$$

In this Eq. 5, *W_HD_* represents the weight of direct sensory information, and *W_HI_* represents the weight of indirect sensory information. Here, the weight of the prior (*W_HP_*) works through the weights of direct and indirect sensory information, as *W_HD_* + *W_HI_* + *W_HP_* = 1. Therefore, the narrower the prior distribution, the larger its relative weight compared with the weights of direct and indirect sensory information [for more details, see Ref. (54)]. In this scheme, the effect of the prior could be seen as the factor that reduces the variance of upright estimates, however, with an accuracy-precision trade-off especially at large tilt angles. Tarnutzer et al. have proposed a Bayesian model to account for the lower SVV precision at larger head tilts based on variability in the otolith inputs. In this model, the preferred directions of the otolith afferents represent different sensitivities to changes in the angle of the head tilt. Thus, an overall likelihood of head position estimate is obtained by combining the probability distributions from individual otolith afferents. In this scheme, the effectiveness of the otolith estimator—reflected by the width of the likelihood distribution—decreases at larger head tilt angles, and it is combined with the prior knowledge of the head orientation to derive the SVV estimate (21).

### Multisensory Contributions

Various studies have addressed contributions of the head, neck, and trunk sensory signals to perception of upright. The findings from these studies indicate that the SVV errors are primarily processed in a head-in-space reference frame (30, 58–62). On the other hand, perception of body orientation is largely modulated by the proprioceptive inputs encoding trunk position, with errors that are more accurate but less precise than SVV responses (54, 55, 63–65). In line with these findings, and consistent with distinct sensory contributions to perception of body orientation from perception of upright, SVV deviations induced by galvanic vestibular stimulation (GVS) were dissociated from the errors in perception of body orientation (66).

In accordance with the multimodal sensory contributions to perception of upright, alterations in the neck, trunk, and interoceptive inputs have modulating effects on perceptual upright responses (30, 61, 67–75). For example, vibration of the neck muscles can shift SVV errors in the opposite direction of the head tilt and increase the E-effect (73, 76, 77). Thus, the brain must be able to determine upright orientation either directly, by accessing the estimate of head-in-space orientation through the sensory inputs encoding head position (e.g., otolith signals), or indirectly, through the sensory inputs encoding neck and trunk positions (54). In this context, the sensory contributions to upright perception are modulated by the body tilt position, with likely a greater weight of the head position signals (e.g., from the otoliths) around the upright position, and a substantial weight of the trunk proprioceptive signals at larger tilt angles (30, 31). Such distinct patterns of sensory contributions to perception of upright are supported by the findings in patients with vestibular and proprioceptive loss (25, 78–87). Patients with vestibular loss tend to have no E-effect at small tilt angles and more pronounced A-effects at larger tilt angles, consistent with reduced weight of head position signals and consequently relative underestimation of upright orientation (25, 80–82, 84, 86, 87). Patients with proprioceptive loss, on the other hand, have decreased A-effect consistent with reduced weight of body proprioception, and consequently relative overestimation of upright orientation (25, 88, 89).

Perception of upright has been also studied with respect to changes in body position or posture (52, 84, 90). Healthy participants lying supine had accurate SVV responses, but there were large errors in patients with vestibular loss in the supine position compared with the sitting and standing positions (84, 91). In general, SVV responses tend to be more accurate while maintaining precarious postures, where there is a risk of falling and thus a higher demand for balancing activity (e.g., standing on a beam) (92, 93). Such findings underscore the ecological aspect of upright perception in which according to the task at hand the internal estimate of upright is modulated by available sensory cues.

Systematic errors of upright perception also occur with body rotation in the roll plane, and—similar to the static roll-tilts—these dynamic errors are dissociated from the perception of the body orientation (27, 94–98). After constant-velocity roll rotations, SVV errors were transiently biased in the direction of the rotation (95–98). This “dynamic” bias was dependent on the velocity of the rotation and the final tilt position at which SVV was measured. For example, with clockwise rotations starting from the upright position, SVV errors showed a significant A-effect when the rotation stopped at large body tilt angles, whereas the errors were close to veridical when it stopped at smaller tilt angles. By contrast, with counterclockwise rotations passing through the upside-down position, SVV errors showed a significant E-effect when the rotation stopped at small tilt angles (i.e., close to the upright position), whereas the errors were close to veridical when it stopped at large tilt angles (i.e., close to the 90° tilt position) (97). This post-rotation “hysteresis” effect lasted about 1 min, suggesting that the transient bias in SVV errors was related to semicircular canal activation from the forces generated through deceleration. Perception of roll-tilt can also be induced during off-axis yaw rotation with the head upright or during on-axis yaw rotation with the head tilted on the body (99, 100). In these scenarios, rotational cues mainly from the horizontal semicircular canal stimulation affect the time course of tilt perception (101, 102). Moreover, SVV errors have been reported with the head pitched forward or backward during yaw-axis rotation. In this case, SVV errors were in the opposite direction of the rotation (same direction as the fast phase of the torsional nystagmus) and were more pronounced with the head pitched backward, consistent with a stronger effect from stimulation of the posterior semicircular canal (103).

Perception of upright has been also studied with respect to the modulating effects of visual backgrounds. Our daily environment is rich with visual cues that indicate world-horizontal and vertical orientations. In general, various visual functions (e.g., orientation discrimination, contrast detection, or visual acuity) show superior performance along the horizontal and vertical axes compared with oblique angles (e.g., 45°), which is referred to as the oblique effect (13, 20). However, visual vertical cues can have a greater effect on one’s perception of spatial orientation than the perceived orientation of objects (104–107). Strong effects of visual cues on upright perception have been shown in various settings, ranging from an entire tilted furnished room to more impoverished stimuli such as a simple square frame (20, 106, 108–119). Remarkably, even the addition of a single line in the SVV paradigm can induce a visual bias in upright responses (118, 120, 121). In the case of a square frame, the visual vertical estimate is biased by the frame orientation, which is known as the rod-and-frame effect. The frame effect can be robust and, for example, significantly decrease SVV errors induced by rotating backgrounds (122, 123). This visual effect for the most part depends on the viewing distance and the head tilt position. It decreases with far viewing, indicating reduced reliability of the frame as a visual cue to upright orientation, and increases with head tilt, indicating reduced reliability of the vestibular cues to upright orientation (119, 124).

**Figure 7:**
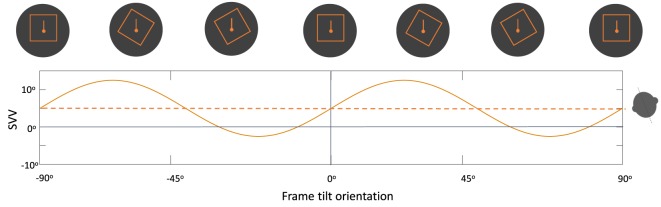
Schematic representation of the periodic subjective visual vertical (SVV) modulation by the frame orientation (solid line) during head tilt: frame tilt orientations close to the subject’s upright perception (dashed line) usually result in an “attractor bias” (i.e., toward the direction of the frame tilt), while there is a “detractor bias” at angles beyond 45° and up to 90° (i.e., away from the direction of the frame tilt). These biases caused by the frame orientation can either attenuate or accentuate SVV errors, depending on head tilt position (e.g., here 20° head tilt to the left).

Overall, changes in the frame tilt orientation can result in periodic modulation of SVV errors by the rod-and-frame effect. Usually, frame tilts close to the perceived upright orientation result in an “attractor bias” toward the frame orientation, whereas there is a “detractor bias” at frame tilts beyond 45° and up to 90°, and no bias at frame tilts close to 90° (Figure 7) (118, 121, 124). This modulating effect of the frame orientation is more pronounced at larger body tilts, and it can either enhance the E-effect or decrease the A-effect depending on the body tilt orientation (105, 118, 125). The rod-and-frame effect may also vary among individuals, as some exhibit a strong frame effect (i.e., visual dependence), while others may have a weaker effect (i.e., visual independence) (126–129). A similar pattern of variability with the rod-and-frame effect has been shown in patients with vestibular loss; however, the frame effect can be asymmetrical in these patients, with reduced or even abolished visual dependence when the frame is tilted toward the healthy side, as opposed to a significant frame effect when it is tilted toward the side of vestibular loss (130). Background rotation in the roll plane (i.e., around the line of sight) can also affect upright perception and induce SVV errors in the direction of the rotation (80, 131, 132). Similar to the rod-and-frame effect, this optokinetic effect is more pronounced at larger body tilt angles and can induce a larger bias toward the side of vestibular loss (83, 133–135).

Another important factor in perception of upright is the effect of gravity on sensory modalities that encode body position (90, 136–141). As a fundamental reference for spatial orientation, the gravity vector plays a significant role in almost all aspects of our balance, perception, and action. In general, gravitoinertial forces can change perceived orientation of objects, an effect that has been described as the oculogravic illusion (142). Similarly, in microgravity and weightless conditions, space crews often report visual reorientation illusions such as difficulty distinguishing between spacecraft floors, walls, and ceiling surfaces (143–146). With respect to upright perception, rotating rooms, parabolic flights, and human centrifuge have been used to study the effects of gravitoinertial forces (39, 101, 102, 140, 147–153). For example, in a centrifuge experiment, perception of tilt significantly increased late in the spaceflight duration compared with the early flight and preflight results on earth (152). This exaggerated perception of tilt also persisted into the early post-flight days. Likewise, other studies using the rod-and-frame test, optokinetic stimulation, and unilateral centrifugations (i.e., stimulating only one labyrinth at a time) have shown significant visual dependency and asymmetry in SVV responses upon returning back to the earth (146, 151, 154). These results suggest that the multisensory contributions to the internal reference for upright orientation is reduced with adaptation to microgravity. The effect of gravity on this multisensory reference is shown with gravitational forces as little as 0.15 *g* (close to the force of gravity at the moon) and up to 1.5–2 *g*, resulting in significant deviations in perception of upright (140, 148, 155, 156).

### Upright Perception and Adaptation: Drift during Head Tilt

**Figure 8:**
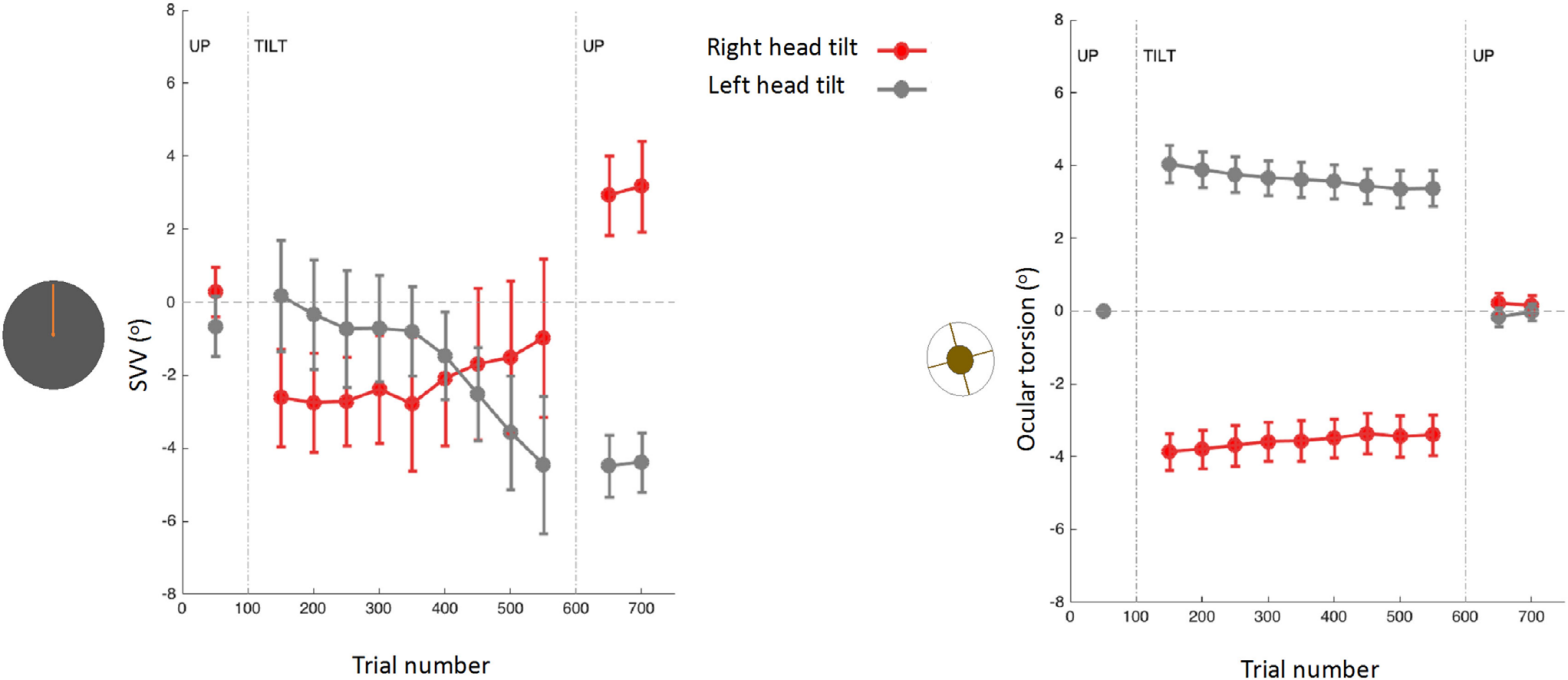
Subjective visual vertical (SVV) and torsional eye position measured simultaneously before, during, and after prolonged head tilts (~15 min) in 12 subjects (15): data points represent SVV or ocular torsion from 100 trials during 20° head tilts to the right and left. Error bars correspond with SEM across subjects. The SVV drift is in the same direction as the head tilt, and when the head returns to upright position there is an aftereffect, also in the same direction as the head tilt. Changes in ocular torsion do not correspond to the SVV drift or aftereffect.

Upright perception may drift during prolonged tilts of the whole body or prolonged tilts of the head on body (15, 31, 61, 157, 158). The drift pattern is usually variable across individuals (157), but often there is a gradual change in the direction of the tilt, followed by a post-tilt bias referred to as the aftereffect (Figure 8) (15, 61, 157–161). When this aftereffect was studied across a wide range of body orientations, there was a “local” effect (as opposed to a “global” effect), where the post tilt bias was mainly seen in the tilt orientations adjacent to the initial, adapting position (162). For example, if the subject was initially tilted at 90°, the SVV aftereffect was more pronounced at nearby tilt angles such as 60°. Based on this finding, it was proposed that maintaining a static tilt position could bias the internal upright reference toward this adopted position, thus resulting in an aftereffect at subsequent tilt positions (162).

As mentioned earlier, ocular torsion can be a significant source of SVV errors during head tilt, due to the low OCR gain and altered orientation of the images on the retina (15, 53, 100, 103). However, neither the drift in upright perception nor the aftereffect correlate with changes in ocular torsion (15) (Figure 8). These findings indicate that the torsional eye position—or its driving input from the otoliths—cannot be the source of the drift or the aftereffect in perception of upright. Similar drifts have been found with haptic measurements, which also confirms that the visual error induced by ocular torsion cannot be the source of drifts in upright perception during head tilt (157, 161). Overall, SVV drifts tend to be larger and more consistent across individuals with the head-on body tilts compared with the whole body tilts (15, 157, 158, 161, 163). These findings, along with predictions from the Bayesian spatial perception model, suggest that the adaptation of neck proprioceptive inputs is the primary source of SVV drift during head tilt (15). Thus, the SVV drift is likely modulated by the position of the head relative to the body rather than the position of the head or trunk relative to gravity. Visual vertical responses may also drift in upright body position, but considerably less when compared with the drift during static body tilt (13, 37). This drift attenuated when upright visual cues were present, but did not completely disappear (13).

## Perception of Upright and Cerebral Cortex

### Multimodal Vestibular Cortex

Multisensory integration is a key functional aspect of neural processes involved in the perception of spatial orientation. In this context, vestibular inputs are often integrated with other sensory modalities that are incorporated into self-perception and extrapersonal spatial orientation to subserve high level cognitive and sensorimotor functions (e.g., visual and proprioceptive signals). Accordingly, graviception and orientation constancy can also be understood as functions mediated by multiple sensory modalities.

Attempts to localize vestibular function to the cerebral cortex began with the ancient descriptions of vertigo and speculations about global cerebral function (164). In recent years, electrophysiological recordings in animal studies have identified multiple cortical sites sensitive to vestibular stimulation, thus laying the groundwork for comparisons with the human cortex. The findings reveal distinct areas within the parietal and temporal cortices that receive and process vestibular inputs. These cortical areas include the parieto-insular vestibular cortex (PIVC), parts of the somatosensory cortex, the lower tip of the intraparietal sulcus, the dorsal subdivision of the middle superior temporal cortex (MSTd), the visual posterior Sylvian area (VPS), and the ventral intraparietal cortex (VIP) [for comprehensive review, see Ref. (165)]. While these vestibular areas are interconnected, there is no clear evidence that they are organized in a hierarchy similar to other sensory regions such as visual and somatosensory cortices. Direct cortical recordings suggest that PIVC is involved in the integration of vestibular and somatosensory information into a concept of “head in space” (166, 167). On the other hand, visual and vestibular signals have been recorded from MSTd, VPS, VIP, and caudal intraparietal area, with reference to heading perception or allocentric orientation in the earth-vertical direction (168–173). Note that despite the evidence for multimodal integration in these cortical areas, vestibular signals recorded from single neurons remain distinct, suggesting that sensory integration takes place through the function of a cortical network rather than individual neurons (174–176).

In human, as with primate studies, findings from cortical lesion analysis, functional imaging with caloric or galvanic stimulation (fMRI and PET), and also direct cortical stimulation point to a widely distributed multisensory vestibular system, mainly in the temporo-partieto-insular cortices [see Ref. (165) for comprehensive review]. The vestibular or combined visual-vestibular activations in these cortical regions are predominantly focused at the temporo-parietal junction (TPJ), and more specifically around the posterior parietal operculum, inferior parietal lobule, superior temporal gyrus (STG), and the junction of the intraparietal sulcus and the postcentral sulcus (177–195). Overall, the patterns of cortical activity in these studies suggest that the posterior parietal operculum is the human homologue of PIVC area in monkey, and the human homologues of VPS, VIP, and MSTd areas are within or around the inferior parietal lobule (180, 196). Note, however, that a systematic mapping of TPJ is currently lacking, and we know little about the flow of sensory information among various areas within this cortical region, or how disruption in one sensory modality may affect multisensory integration and perception of spatial orientation.

Although not addressed in animal studies, significant vestibular activation has been found in the non-dominant human cortex, i.e., the right hemisphere in right-handers and the left hemisphere in left-handers (179). Notably, the cortical mechanisms involved in spatial functions also modulate lower-level vestibular function, and a similar pattern of laterality has been shown for the cortical influence on the duration of the vestibulo-ocular reflex (i.e., the time constant) (197–199). With respect to the vestibular connections to the cerebral cortex, five distinct vestibular pathways have been identified based on functional and structural imaging analyses (200, 201). Three of these pathways run ipsilaterally, and two cross either within the pons or the midbrain. The ipsilateral pathways reach the inferior part of the insular cortex either directly or through the thalamus. Contralateral pathways run through the posterolateral thalamus to the parieto-insular cortex. In addition to connections with the brainstem, the parietal opercular regions also maintain communication with each other via an interhemispheric band of fibers passing through the antero-caudal splenium of the corpus callosum (200, 201).

### Temporo-Parietal Cortex and Perception of Upright

The TPJ is a cortical hub for multiple sensory modalities, and it has been implicated in various aspects of spatial orientation including visuospatial attention, heading perception, visual gravitational motion perception, sense of embodiment, self-localization, and egocentricity (186, 187, 191, 202–213). The role of TPJ in perception of spatial orientation is especially evident from the deficits in neglect syndrome as a result of lesions involving this cortical region. Patients with neglect are unable to attend to sensory stimuli in their contralesional hemispace and also show significant contraversive deviations of upright perception in both haptic and visual tasks (214–223). These multimodal deficits in upright perception are often related to the severity of neglect symptoms and are also modulated by the head and body positions (217, 220, 224–228). In addition, abnormal visual modulation of upright perception has been reported in neglect patients. Using the rod-and-frame test, upright responses were more biased by the frame effect when it was tilted contralesionally, whereas the bias decreased when the frame was tilted toward the side of the lesion (216). Visuospatial deficits (i.e., visual extinction) have been also produced in healthy individuals by the inhibitory effect of transcranial magnetic stimulation (TMS) over the right TPJ. This transient effect, as with neglect patients, was dependent on the horizontal and vertical eccentricity of the visual stimulus (229). Taken together, these findings suggest that the perception of body orientation, visuospatial awareness, and upright orientation share the same cortical networks. In this scheme, sensory processing at the TPJ would be crucial for construction of the reference frames used for both self-position and extrapersonal space transformations. In line with the multisensory role of TPJ, cortical activations within this area during visual, tactile, and vestibular sensory conflicts correspond to the perception of self-location (230–232). Accordingly, TPJ lesions are also associated with symptoms such as out-of-body experience or room tilt illusion (210, 231, 233–236). Overall, these lines of evidence indicate that TPJ is involved in generating the multisensory internal reference used by the brain to anchor “self” with respect to the surrounding environment and maintain orientation constancy especially with changes in the eye, head, and body positions.

**Figure 9:**
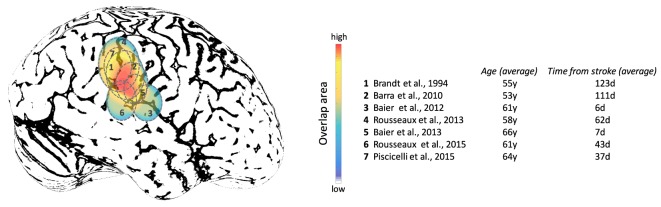
Approximate projections of the cortical areas associated with subjective visual vertical deviation based on anatomical locations of the average lesion areas from seven studies. The color map shows the degree of overlap among cortical involvement in these studies with maximum convergence around the temporo-parietal junction. The average age of the patients in years and the average time from the stroke in days are included for each study.

Studies focused on the effects of brain lesions on upright perception go back as far as 1948, where SVV errors exceeding 2° were described with fronto-parietal lesions, but not occipital lesions (for comparison, note the campanile of Pisa is currently at 4°) (237). More recently, lesion studies have shown associations between cerebral cortex and abnormal upright perception in the context of hemispheric stroke (88, 221, 226, 238–242). Note that these studies have recruited patients at different post-lesion times which could affect the SVV results depending on the effect of brain adaptation following the stroke in these patients. While these studies indicate involvement of several cortical areas within and around TPJ, these lesions converge largely within the inferior parietal lobule and posterior aspect of the insular cortex (Figure 9). Isolated lesions within the posterior insula, however, are not associated with SVV deviations, which suggests that other cortical locations within TPJ are involved in perception of upright (243). With respect to subcortical white matter regions, lesion extensions to the superior longitudinal fascicle, inferior longitudinal fascicle, inferior occipitofrontal fascicle, and superior occipitofrontal fascicle are shown in connection with SVV deviations (239, 242). In general, lesion studies have widely reported contralesional SVV deviations, whereas only about 10% of patients may have ipsilesional SVV deviations (88, 220–222, 228, 237, 238, 240, 241, 244–248). This finding contrasts with to the SVV deviations seen with brainstem lesions, which more consistently are tilted toward the side of the lesion with caudal brainstem involvement, and away from the side of the lesion with rostral brainstem involvement (249–251). In addition, the extent of SVV deviations with cerebral cortical lesions is usually less than the SVV deviations with the brainstem or peripheral vestibular lesions (251, 252). These anatomical differences in SVV errors are likely related to the pathological changes in ocular torsion with low-level brain lesions. Such deviations in ocular torsion lead to SVV errors by directly affecting the orientation of the images on the retina. SVV errors at the level of cerebral cortex, on the other hand, are primarily linked to the neural sensory processes underlying spatial perception.

Generally, SVV errors from the right hemispheric lesions tend to be larger, long lasting, and more often associated with contralesional deviations (239, 245, 247, 248). These findings are consistent with the dominance of the right hemisphere in processing spatial information. In addition, the magnitude of SVV deviations correlates with the extent of cortical lesions, highlighting the significance of a multisensory cortical network for coherent perception of upright (88, 247). The contralesional SVV bias persists with small body tilts away from the side of the lesion, resulting in an A-effect toward the paretic side, instead of a normal E-effect in the opposite direction (88, 220, 228, 244). Such bias, however, is not present when the body is tilted toward the side of the lesion (i.e., away from the paretic side), in which case the SVV errors are comparable to normal individuals (88). It is also shown that the errors of upright perception from cortical lesions could be dissociated from perception of body position or actual postural deviations. However, patients with concurrent errors in all these domains had lesions involving the right TPJ (247, 253, 254). When measured at different body tilts, SVV and perception of body position were correlated when the body was tilted toward the side of the lesion, but such correlation was not present while tilted away from the side of the lesion (244, 255). There were also larger overestimation errors in perception of body position compared with SVV while the body was tilted away from the side of the lesion. Such dissociation between perceptions of upright and body position is consistent with different weights of sensory contributions for processing upright orientation versus body position. With respect to other axes of spatial perception, a significant backward deviation of upright responses in the pitch plane has been reported in patients with right hemisphere stroke in addition to the errors in the roll plane (222, 223).

**Figure 10:**
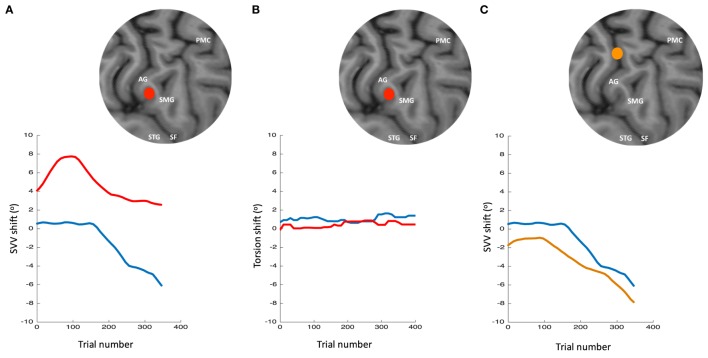
Simultaneous subjective visual vertical (SVV) and ocular torsion recordings during prolonged left head tilt of 20° in a single subject (500 trials ~15 min) [data from Ref. (260)]: SVV shift from transcranial magnetic stimulation (TMS) at SMGp (red) is shown along with the SVV shift from the sham stimulation (i.e., no TMS) (blue) **(A)**. In both traces, there is a gradual drift over time toward the left (i.e., in the same direction as the head tilt), but the SVV shift from TMS is larger with a deviation opposite to the direction of the head tilt. Ocular torsion shift from TMS at SMGp (red) is not different form the sham stimulation (blue) **(B)**. As opposed to SMGp, SVV shift from TMS at another cortical location outside of TPJ (orange) is smaller than the sham stimulation with a deviation in the same direction as the head tilt **(C)**. PMC, primary motor cortex; SMG, supramarginal gyrus; AG, angular gyrus; STG, superior temporal gyrus; SF, Sylvian fissure.

The role of TPJ in perception of upright is also studied using non-invasive brain stimulation (256–259). We recently applied TMS in healthy participants at the right TPJ and probed its transient cortical effects on perception of upright using SVV measurements (256). The inhibitory effect of TMS at the posterior aspect of the right supramarginal gyrus (SMGp) resulted in a shift of SVV errors in the opposite direction of the head tilt (Figure 10). The direction of this error, induced by the focal cortical inhibition, is consistent with the “overestimation” errors reported by the cortical lesion studies [i.e., increase in E-effect; e.g., Ref. (88)]. On the other hand, when TMS was applied randomly at other cortical locations within or outside of the TPJ, there was no significant SVV deviation, suggesting a location-specific effect at SMGp. In addition, there was no change in the torsional position of the eyes despite the SVV shift at SMGp, showing that the changes in perception of upright at the level of cerebral cortex were dissociated from the changes in ocular torsion (260) (Figure 10). Altogether, these findings suggest that unlike subcortical regions that have direct influence over ocular torsion, TPJ is primarily involved in sensory processing. Fiori et al. also investigated the role of TPJ in upright perception using the focal inhibitory effects of TMS (257). They found that the effect of TMS at the right TPJ selectively increased SVV errors when no visual cue was provided (i.e., no visual frame during the SVV task). However, inhibition of V1–V3 and not TPJ disrupted the visual detection of a Gabor patch orientation. This functional distinction between TPJ and early visual cortex is in line with the role of TPJ in multisensory integration for perception of upright. A significant SVV shift has also been shown using transcranial direct current stimulation (tDCS) over TPJ (258). This shift was dependent on tDCS electrode placement, with SVV deviation toward the side of anode placement. There was also a rebound effect (i.e., reversal of the SVV shift) immediately after the stimulation, which lasted longer with the right cathode/left anode placement. Cortical involvement in perception of upright has also been investigated using EEG recordings (261, 262). The results suggest that early cortical activity in the lateral temporo-occipital cortex (around 100 ms post-stimulus) is important for extracting orientation features, whereas a later activation involving the temporo-occipital and parieto-occipital cortices (around 300 ms post-stimulus) reflects multisensory integration for perception of upright.

Peripheral vestibular injuries can also provide clues to the mechanisms of recovery and multisensory compensation with respect to cortical function and upright perception. For example, it is reported that hemispheric dominance can affect the speed of recovery based on the side of peripheral vestibular injury. The recovery from the right-side vestibular loss was significantly slower than from the left-side vestibular loss in right-handers, while such difference was not found in left-handers (87). Based on this observation, it was hypothesized that the difference in the pattern of recovery in left-handers is related to a greater distribution of transcallosal connections between parietal cortices compared with right-handers (87).

### Cerebral Cortical Pathology and Perception of Upright

Pathological perception of upright is widely reported with cerebral infarctions (88, 214, 220–222, 226, 228, 238–240, 244–247, 249, 253, 255, 263–285). SVV deviations in association with cortical strokes are typically found in the territory of the medial cerebral artery (MCA), mainly involving the temporal, parietal, and insular cortices. The absence of skew deviation of the eyes with these lesions suggests the affected cortical areas are primarily involved in processing sensory information (238). Notably, posterior cerebral artery infarctions, despite causing visual field defects, do not significantly alter perception of upright (238). In a sample of unilateral hemispheric infarction, the branches of the MCA resulting in SVV deviation were the temporal (mean SVV deviation about 6°), parietal (mean SVV deviation about 5°), and the deep cortical perforators (mean SVV deviation about 4°). Lesions affecting the anterior part of the internal capsule can also be associated with SVV tilt (mean SVV deviation about 3°), primarily via the lenticulostriate arteries and the anterior choroidal artery (238).

In general, hemispheric infarcts more often result in contraversive SVV deviations, while about 10% of patients may show ipsiversive SVV deviations. Pathological SVV tilts can be as large as 15°, though usually they are 5–10° and deviated leftwards as a result of right hemispheric lesions (note again that the campanile of Pisa is currently at 4°) (238, 275, 286). The range of SVV deviations in a sample of 40 patients with hemispheric stroke (time from lesions <13 weeks) was larger with the right hemispheric infarcts (−13.1° to 3.2°) compared with the left hemispheric infarcts (−3.6° to 9.3°) (228). The asymmetric hemispheric contribution to upright perception has been also shown in stroke patients with the bottom-up effects of GVS (276, 287). In these patients with right hemispheric infarcts and spatial neglect, left-cathodal but not right-cathodal galvanic stimulation significantly reduced SVV deviations, highlighting a significant cortical laterality for perception of upright. Another important factor affecting the extent and direction of SVV errors is the recovery time. Acute patients often have larger SVV errors compared with chronic patients, and such deviations often recover significantly within a few months (239, 245, 286). Patients with right hemispheric lesions also have higher variability (i.e., lower precision) in their SVV deviations (286).

Persistent SVV errors and low SVV precision are often linked to poor balance following stroke, especially in patients with the right hemispheric involvement (263, 286, 288, 289). However, perception of body orientation can be dissociated from SVV or from the actual postural deviations in these patients (63, 244, 247, 249, 253, 255, 273, 275, 277, 280–282, 290–294). For example, in a sample of 80 stroke patients reported by Perrenou et al., 34 had abnormal contralesional postural vertical tilts (i.e., deviations in posture alignment with perceived upright orientation), 44 had contralesional SVV tilts, 26 had contralesional haptic vertical tilts, and none had ipsilesional haptic or postural vertical tilts (247). Forty-one patients (52%) showed deficits in more than one modality, and 18 (22%) had transmodal contraversive deviations (i.e., SVV, postural vertical, and haptic vertical were all tilted away from the side of the lesion). In general, postural deviations in stroke patients are more closely related to the errors of postural vertical perception than to the errors of upright perception (220, 244, 247, 254, 286).

**Figure 11:**
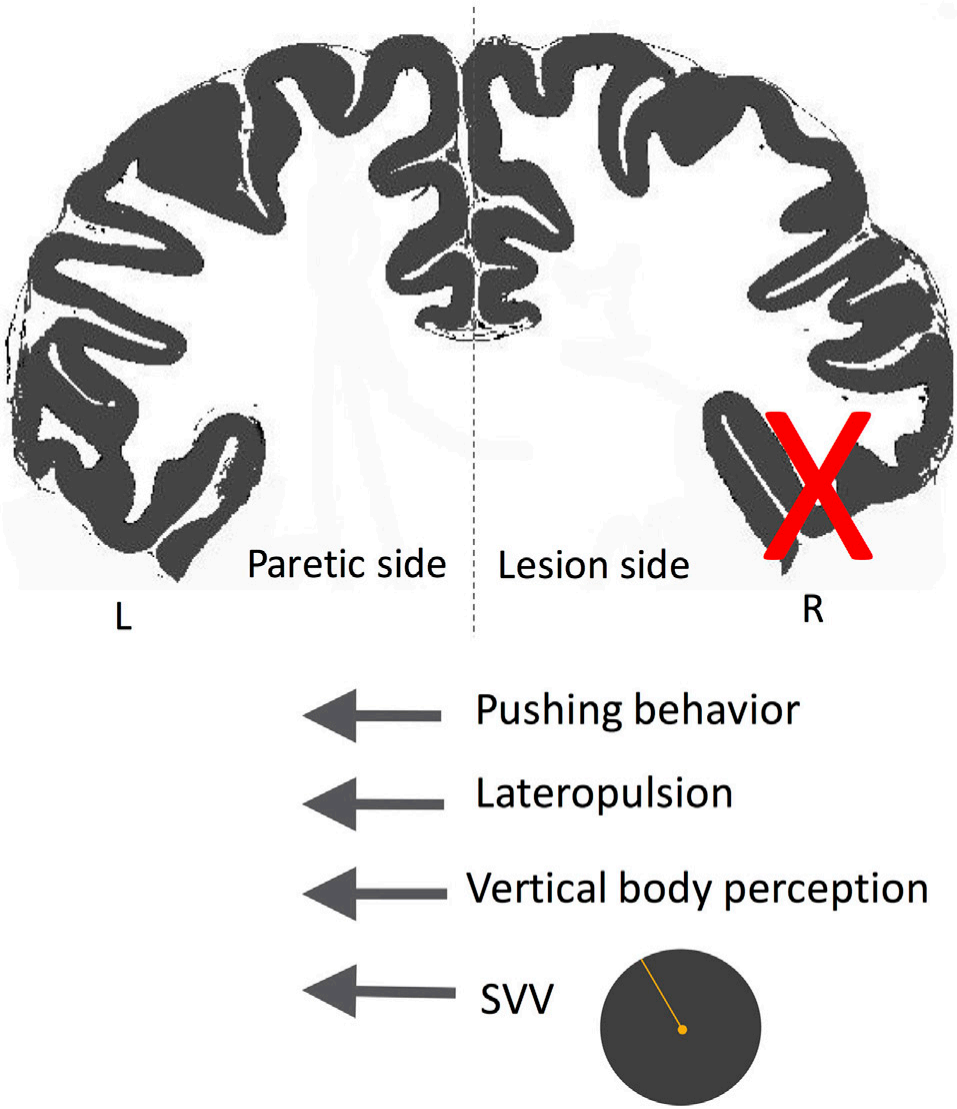
Schematic showing the directions of subjective visual vertical (SVV) tilt and postural deviation in pusher syndrome. In these patients, SVV and postural vertical perception deviate away from the side of the lesion (X), matching the direction of postural tilt (i.e., lateropulsion) as well as the pushing behavior toward the paretic side. Therefore, patients with pushing behavior seem to actively align their body with erroneous upright and postural estimates.

A subset of patients with cortical infarctions and postural deviations exhibit robust SVV deviations and also actively resist attempts to correct their false postural orientation back to upright position (247, 253, 265, 266, 270, 272, 274, 277, 281, 284, 294–300). This phenomenon, referred to as “pusher syndrome” (also “listing,” or “lateropulsion”), is typically toward the paretic side with an incidence of approximately 5–10% among acute stroke patients (266, 278, 288). In contrast to patients with Wallenberg syndrome or thalamic astasia who pull themselves back toward upright to prevent an ipsilesional fall, pushers resist postural changes toward the non-paretic side. Patients with pusher behavior are often unable to learn to walk even with proper assistance, and their SVV errors or postural vertical deviations often last longer (275). Pushing behavior is also highly correlated with neglect symptoms and more often is associated with lesions involving the right posterior insula, STG, inferior parietal lobule, and postcentral gyrus (247, 253, 274, 278, 279, 281, 288, 297, 301, 302). In the study of Perennou et al. mentioned earlier, the patients who showed lateropulsion and pusher behavior had contraversive transmodal tilt of postural vertical, haptic vertical, and SVV (247). This finding suggests that lateropulsion and pushing behavior lie on a continuum where pushers—as opposed to those with lateropulsion only—actively align their body with their erroneous perception of upright (Figure 11) (247, 280, 299). When postural vertical perception was measured while standing (as opposed to sitting in other studies), pushers had large uncertainty, and, on average, ipsilesional deviation in their responses (303), showing that the postural vertical estimates can be altered by active pushing behavior while standing.

Parkinson’s disease (PD) is another pathology that can affect postural control and spatial perception, due to dysfunctions involving the cortical connections with basal ganglia (304, 305). On this premise, PD measures such as trunk flexion, stance, and gait parameters have been investigated in association with SVV deviation (306–311). The postural instability in PD patients may correlate with SVV deviations, and both postural vertical perception and SVV show higher variability compared with age-matched, healthy controls (312, 313). In these patients, however, visually induced postural sway cannot be linked to the deficits in perception of upright, which suggests that the postural instability is related to abnormalities in maintaining posture rather than perceptual errors (80, 314). PD patients may also have trunk lateropulsion with the tendency for postural tilts in the direction opposite to the affected side of the body (once dubbed “scoliosis of Parkinsonism”) (315). The patients with lateral trunk deviation show significantly larger SVV errors toward the trunk tilt compared with those without trunk tilt (308, 310). This lateral trunk tilt in PD has been attributed to vestibular hypofunction on the same side and described as postural imbalance syndrome with vestibular alterations or PISA (311). Patients with PISA have greater SVV deviations compared with those without the trunk tilt, either on or off of the effects of dopaminergic medications (310). Taken together, the above findings suggest that abnormal upright perception in PD patients can be linked to impaired sensorimotor processing related to corticobasal dysfunction.

Migraine syndrome can also result in visuospatial symptoms due to dysfunctions affecting neural networks from the level of brainstem to the cerebral cortex. Migraine patients with these symptoms typically complain of vertigo, dizziness, disorientation, or sense of disequilibrium, often triggered or worsened with changes in the head or body positions. This type of migraine presentation accounts for the most common cause of episodic dizziness and is classified as vestibular migraine (316–319). Patients with vestibular migraine have more pronounced postural sway compared with other types of migraine or healthy controls (320, 321). Consistent with the visuospatial symptoms in these patients, imaging analyses have found decreased gray matter volume within TPJ as well as metabolic changes in this cortical region during the attacks of vestibular migraine (322, 323). With respect to upright perception, several studies have reported SVV measurements in migraine patients (322–327). According to these studies, patients with non-vestibular migraine correctly estimate upright orientation, while those with vestibular migraine show higher variability in SVV errors compared with other headache disorders or healthy controls (319, 327–330). Patients with vestibular migraine also have reduced motion detection thresholds in the roll plane compared with non-vestibular migraine or healthy controls (331). However, currently, it is not known whether these patients with vestibular migraine also have altered perception of upright during static head or body tilts.

## Summary and Conclusion

As a multimodal sensory reference, perception of upright represents neural processes that subserve orientation constancy. Consistent with the multisensory properties of these neural processes, several studies have described modulatory effects of gravity, visual cues, and position of the body on perception of upright. Also, various measurement paradigms have shown systematic errors of upright perception with tilting the head or body (i.e., underestimations of the true vertical orientation at large tilts and overestimations at small tilts). These errors reflect challenges for the brain in maintaining a common reference frame for upright orientation, based on the reliability of sensory signals that encode head, eye, and body positions. The computational mechanisms behind these systematic errors have been addressed using mathematical models that account for noisy sensory signals. In these models, the estimates of head, body, and ocular torsion that determine upright orientation are derived using frameworks such as Bayesian “prior” and relative weighting of sensory information.

Concerning the role of cerebral cortex in various aspect of spatial perception, animal and human studies show a widely distributed cortical network, primarily within the temporal, insular, and parietal cortices. This is not surprising considering the vital role of the information about body orientation with respect to the surrounding environment while any motor action is being contemplated. With respect to upright perception, the higher-order neural mechanisms must solve the problem of different sensory reference frames in the process of integrating various sensory information. The evidence for cortical involvement in such neural processes comes from TMS and lesion studies. The inhibitory effect of TMS at the posterior aspect of the supramarginal gyrus results in overestimation of upright orientation in the opposite direction of the head tilt. Likewise, cortical lesions involving TPJ are associated with SVV deviations primarily away from the side of the lesion. Patients with these cortical lesions may also have neglect symptoms or out-of-body experiences. Altogether, these findings suggest that perception of body orientation, visuospatial awareness, and upright orientation share the same cortical networks in which an internal reference is generated to anchor “self” with respect to the outside world and maintain orientation constancy. Currently, little is known about the flow of sensory information within these cortical networks and how disruption of one sensory modality may affect processing or integration of other sensory modalities. Future studies will have to specifically address such sensory contributions with respect to cerebral cortical involvement in perception of upright.

## Author Contributions

Both authors have contributed to the data gathering and writing of this manuscript.

## Conflict of Interest Statement

The authors declare that the research was conducted in the absence of any commercial or financial relationships that could be construed as a potential conflict of interest.
